# Epidemiology of first- and second-line drugs-resistant pulmonary tuberculosis in Iran: Systematic review and meta-analysis

**DOI:** 10.1016/j.jctube.2024.100430

**Published:** 2024-03-16

**Authors:** Sara Abbasian, Hamid Heidari, Danyal Abbasi Tadi, Jalil Kardan-Yamchi, Asieh Taji, Atieh Darbandi, Parisa Asadollahi, Abbas Maleki, Hossein Kazemian

**Affiliations:** a Laboratory Sciences Research Center, Golestan University of Medical Sciences, Gorgan, Iran; b Department of Microbiology, Faculty of Medicine, Shahid Sadoughi University of Medical Sciences, Yazd, Iran; c Department of Veterinary, Azad University of Shahr-e Kord, Shahr-e Kord, Iran; d Quality Control and Screening Management Office, Deputy of Technical and New Technologies, Iranian Blood Transfusion Organization, Tehran, Iran; e International Campus, Shahid Sadoughi University of Medical Sciences, Yazd, Iran; f Department of Microbiology, Faculty of Medicine, Iran University of Medical Sciences, Tehran, Iran; g Clinical Microbiology Research Center, Ilam University of Medical Sciences, Ilam, Iran

**Keywords:** Tuberculosis, Drug resistance, First- and second-line drugs, Iran, Systematic review and meta-analysis

## Abstract

Drug resistance among *Mycobacterium tuberculosis* (MTB) strains is a growing concern in developing countries. We conducted a comprehensive search for relevant studies in Iran on PubMed, Scopus, and Embase until June 12, 2020. Our study focused on determining the prevalence of antibiotic resistance in MTB isolates, with subgroup analyses based on year, location, and drug susceptibility testing (DST) methods. Statistical analyses were performed using STATA software. Our meta-analysis included a total of 47 articles. Among new TB cases, we found the following prevalence rates: Any-resistance to first-line drugs: 31 % (95 % CI, 24–38), mono-drug resistance: 15 % (95 % CI, 10–22), and multidrug resistance to first-line drugs: 6 % (95 % CI, 4–8). There was a significant variation in the rate of MDR among new TB cases based on the year of publication, location, and DST methods (*P* < 0.0001). We observed substantial variability in multidrug-resistant TB rates among new cases across the studies. Stratified analyses revealed that publication years and DST methods significantly affected resistance rates. Studies from southern and central Iran reported higher any-drug resistance rates, suggesting regional differences. Among retreatment cases, the prevalence rates were as follows: Any resistance: 68 % (95 % CI 58–78), mono-resistance: 19 % (95 % CI 7–34), multidrug resistance: 28 % (95 % CI 15–43). Our study revealed that the prevalence of drug-resistant TB (DR-TB) among TB cases in Iran is higher than the global average. Particularly, MDR-TB among retreatment TB cases is a significant public health issue.

## Highlights

• Our *meta*-analysis included a total of 47 articles.
• The prevalence rates in new TB cases: Any-resistance to first-line drugs: 31 % (95 % CI, 24–38), mono-drug resistance: 15 % (95 % CI, 10–22), and multi-drug resistance to first-line drugs: 6 % (95 % CI, 4–8).
• There was a significant variation in the rate of MDR among new TB cases based on the year of publication, location, and DST methods (*P* < 0.0001).
• The prevalence rates in retreatment cases: Any resistance: 68 % (95 % CI 58–78), mono-resistance: 19 % (95 % CI 7–34), multi-drug resistance: 28 % (95 % CI 15–43).

## 1 Introduction

Globally, tuberculosis (TB) is the second leading infectious killer after *Coronavirus* disease (COVID-19) worldwide [1], [2]. The continuing spread of drug-resistant TB is one of the biggest challenges and concerns [3]. Identifying high-risk areas is crucial for each country to achieve the ultimate goal of eliminating TB by 2050. Accurate and timely detection of resistance to second-line drugs is of paramount importance to optimize treatment and direct infection control measures to block the transmission of multidrug-resistant TB (MDR-TB) and minimize the risk of further resistance development. The World Health Organization (WHO) suggests that all presumptive TB patients undergo drug susceptibility testing (DST), although numerous countries still lack the laboratory capacity for this [4]. Thus, in many low- and middle-income countries, there may be a high level of under-diagnosis and misdiagnosis of drug-resistant TB (DR-TB). Both phenotypic and genotypic DST methods are employed for susceptibility testing to anti-TB drugs [5]. The proportion method, a phenotypic DST method, is widely used in developing countries and low-income countries like Iran. Therefore, we have stratified a subgroup based on DST methods (proportion versus proportion methods plus other methods).

TB remains a significant public health concern in developing countries such as Iran. However, TB incidence in Iran showed a decreasing trend in 2020 (13 cases per 100,000 populations), with a treatment success rate of 85 % among new and relapse cases registered in 2019.

Two systematic review and *meta*-analysis studies [6], [7] on TB drug resistance have already been published in Iran. However, these published studies lack the most detailed data on TB drug resistance patterns in new or retreated TB cases. Therefore, our comprehensive *meta*-analysis was conducted to evaluate the weighted pooled resistance rate (WPR: proportion of strains resistant to specific antimicrobial agents) in different drug resistance statuses (any drug, mono drug, MDR, pre-extensively drug-resistant (XDR), XDR, and also first-line (isoniazid, rifampicin, streptomycin, ethambutol, pyrazinamide, HRES (isoniazid + rifampicin + streptomycin + ethambutol), HRE (isoniazid + rifampicin + ethambutol), HRS (isoniazid + rifampicin + streptomycin), and HR (isoniazid + rifampicin)) or second-line anti-TB drugs (amikacin, kanamycin, ethionamide, ofloxacin, capreomycin)) in MTB isolates. We also conducted subgroup analyses by year (2000–2015, 2016–2020), location (North, Center, East, West, South), and DST methods (proportion method and proportion method plus other methods) in new and retreated pulmonary TB cases.

The results of our review will provide a more comprehensive understanding of the current epidemiology of drug-resistant pulmonary TB in Iran. This knowledge will promote the development of more effective antimicrobial stewardship programs to combat, control, manage, and limit the development of these drug-resistant organisms.

## 2 Methods

### 2.1 Guidelines, data sources and search strategy

This review follows the Preferred Reporting Items for Systematic Reviews and Meta-Analyses guidelines (PRISMA) [8]. A comprehensive systematic literature search was conducted in three databases: MEDLINE [PubMed], Scopus, Web of Science, and Embase for relevant articles (up to June 12, 2020). The search used the following terms: (“*Mycobacterium tuberculosis*” OR “M. tuberculosis” OR “tuberculosis” OR “TB”) AND (“drug resistance” OR “drug susceptibility” OR “Anti-tuberculosis resistance” OR “anti-TB resistance”) AND (“Iran”) in the Title/Abstract/Keywords fields.

### 2.2 Inclusion and exclusion criteria

Studies providing sufficient original data on the prevalence of first (isoniazid, rifampin, ethambutol, streptomycin, pyrazinamide) or second (fluoroquinolones, aminoglycosides, ethionamide, capreomycin) line anti-TB drugs and reporting the status of drug resistance in pulmonary TB in Iran were included. Drug-resistance data for either new or retreated cases, or both were also incuded. If the study was reported in duplicate, the first published version was included.

Animal research, reviews, congress/conference abstracts, *meta*-analyses, or systematic reviews, duplicate publication of the same study, and articles available only in abstract form were excluded. Moreover, studies reported in languages other than English, articles focused solely on extrapulmonary TB or TB cases coinfected with human immunodeficiency viruses (HIV) or childhood TB, studies that have not performed or reported DST were excluded. To minimize potential bias due to small sample size, articles with < 10 cases were also excluded.

### 2.3 Data extraction and definitions

Two authors independently extracted data from the included studies. The information extracted from each study included first author, year of publication, study period, province, distribution of age and gender in the population, sample size of cases, number of isolates, and drug resistance status (any drug, mono drug, MDR, pre-XDR, XDR, and also first or second-line anti-TB drugs).

Resistance among new TB cases is defined as a newly registered episode of TB in a patient who has never been treated for TB or has taken anti-TB medicines for less than one month. Resistance among retreatment TB cases refers to patients who have received one month or more of anti-TB medicines in the past [9], [10]. Mono-resistance is defined as resistance to only one first-line anti-TB drug. MDR represents resistance to at least isoniazid and rifampin. Any drug resistance is defined as resistance to any drug regardless of mono-resistance or MDR [9], [10]. Pre-XDR is defined as TB caused by MTB strains that fulfill the definition of MDR and rifampicin-resistant TB (RR-TB) and are also resistant to fluoroquinolone [9], [10]. XDR is defined as TB caused by MTB strains that fulfill the definition of MDR/RR-TB and are also resistant to any fluoroquinolone and at least one additional Group A drug [9], [10].

### 2.4 Quality assessment

The quality of the included studies was assessed independently by two reviewers using an adapted version of the Newcastle-Ottawa assessment scale adapted for cross-sectional studies [11]. A score ranging from 0 to 7 points was attributed to each study (7 points: high quality, ≤ 6 points: low quality).

### 2.5 Statistical analysis

To analyze and combine the results of different studies, the prevalence of resistance in each study was considered as a binomial distribution, and its standard error was calculated accordingly. Heterogeneity among studies was assessed using Cochran's Q test, I^2^ index, and interval. Due to the heterogeneity of the studies, a random-effects model was used in the *meta*-analysis. Sensitivity analysis was performed to evaluate the sources of heterogeneity between studies. The analysis was conducted using Stata/SE software, v.14 (StataCorp, College Station, TX). Publication bias was analyzed using Egger’s linear regression test. All statistical interpretations were reported with a 95 % confidence interval (CI).

### 2.6 Study outcomes

The primary outcome of the study was the weighted pooled prevalence of drug resistance status (any drug, mono drug, MDR, pre-XDR, XDR, and first or second-line anti-TB drugs) in MTB isolates. Subgroup analyses were performed based on (1) DST method (proportion method vs proportion methods plus other methods [PCR-based sequencing methods or minimal inhibitory concentration (MIC)-based methods or GeneXpert or line-probe assays or sequencing]), (2) year of publication (2000–2015, 2016–2020), and (3) geographical areas (North, Center, East, West, South).

## 3 Results

### 3.1 Characteristics of studies

**Fig. 1:**
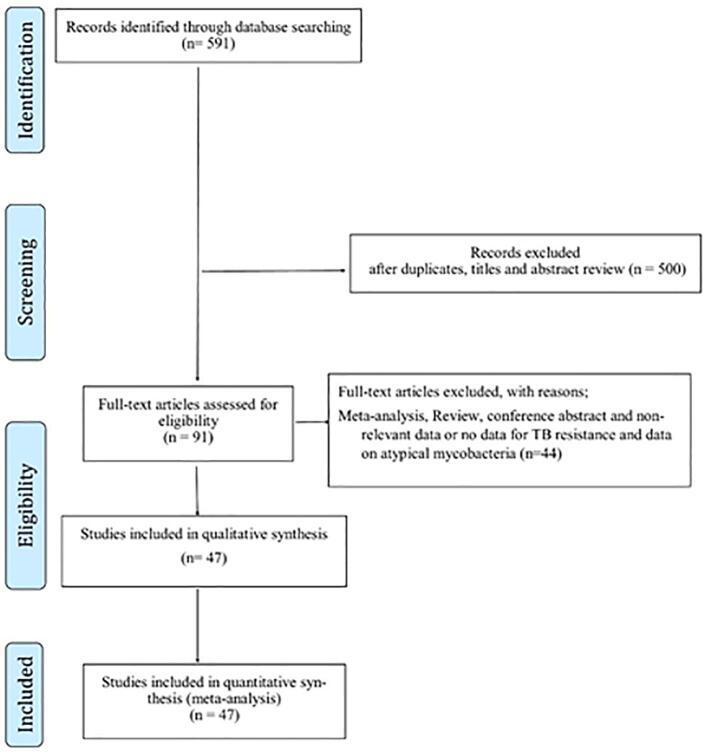
Flow Diagram Showing the Study Selection Process.

Fig. 1 illustrates the study selection process. Initially, 591 studies were identified during the early literature search. Following the initial screening, 500 articles were excluded due to duplication, irrelevance based on title and abstract. The full texts of the remaining 91 articles were carefully reviewed. Among these, 44 articles were excluded based on predefined exclusion criteria. Finally, 47 eligible articles [12], [13], [14], [15], [16], [17], [18], [19], [20], [21], [22], [23], [24], [25], [26], [27], [28], [29], [30], [31], [32], [33], [34], [35], [36], [37], [38], [39], [40], [41], [42], [43], [44], [45], [46], [47], [48], [49], [50], [51], [52], [53], [54], [55], [56], [57], [58] were included in the *meta*-analysis, providing valuable information on first-line anti-TB drug resistance among new and retreatment cases, as well as data on second-line anti-TB drug resistance among new cases.

The majority of the included studies were conducted in the central region of Iran (n = 16), followed by the southern region (n = 12), western region (n = 6), eastern region (n = 2), northern region (n = 2), and various locations across Iran (n = 9). In terms of drug susceptibility testing (DST) methods, 33 studies exclusively utilized the proportion method, while 13 studies employed a combination of proportion methods and PCR-based sequencing methods, minimal inhibitory concentration (MIC)-based methods, GeneXpert, line-probe assays, or sequencing. Additionally, one study (53) employed a PCR-sequencing assay. Diagnosing drug resistance TB preferably carried out in reference laboratories that are subject to rigorous and standardized quality assurance measures.

### 3.2 Study population

The analysis encompassed a total of 12,492 TB patients, comprising 11,492 new TB cases and 1,000 previously-treated TB cases.

### 3.3 First-line Anti-TB drug resistance among new and retreatment cases

For new cases, resistance was most common against streptomycin (18 %), followed by isoniazid (12 %), rifampin (11 %), pyrazinamide (10 %), and ethambutol (8 %). The prevalence of any-drug resistance, mono-drug resistance, and multidrug-resistant TB (MDR) among new cases was found to be 31 % (95 % CI, 24–38), 15 % (95 % CI, 10–22), and 6 % (95 % CI 4–8), respectively.

**Fig. 2:**
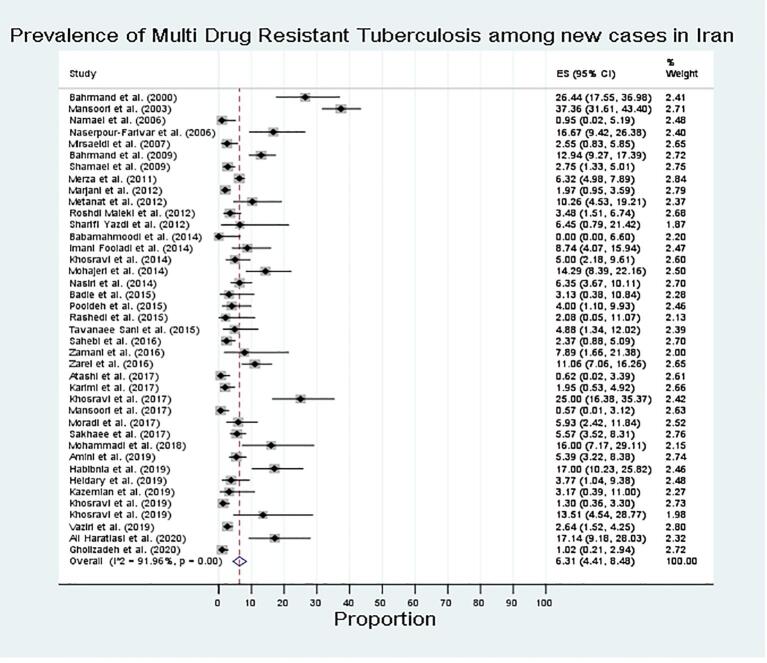
Prevalence of Multi Drug Resistant TB among new cases in Iran.

**Fig. 3:**
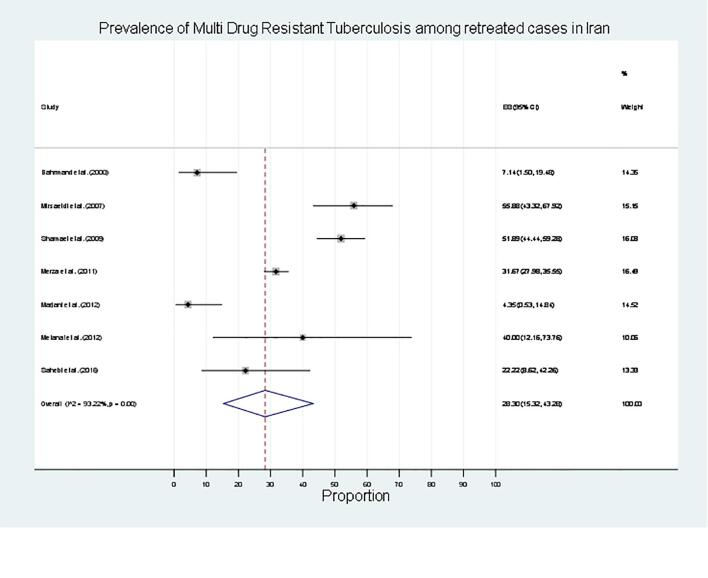
Prevalence of Multi Drug Resistant TB among retreated cases in Iran.

**Fig. 4:**
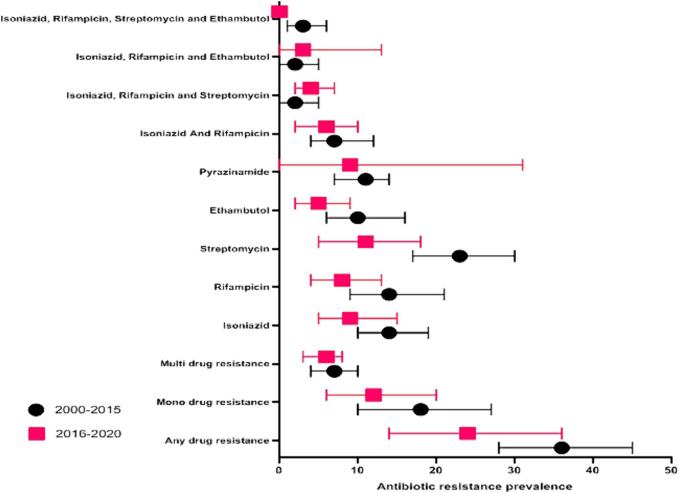
Prevalence of first line drug TB among new cases in Iran based on published year.

Fig. 2, Fig. 3 show the forest plots of the *meta*-analysis of MDR in new and retreatment TB cases. There was a significant variation in the rate of MDR among new TB cases among the included studies (P < 0.0001; Fig. 4). Stratified analyses revealed significant differences between published years and DST methods in pooled rates of any-drug resistance, mono-drug resistance, first-line anti-TB drug resistance, and MDR (Table 1; P < 0.0001). Table 1 displays the stratified location of the *meta*-analysis in new TB cases. Higher rates of any-drug resistance TB were observed for studies from the south and center of Iran.

**Table 1:** Prevalence of Drug-Resistant TB Among New Cases in Iran.

Variables	Sub-groups	Number of studies	Prevalence of Drug Resistance (95 % CI)	n, N	Heterogeneity Test (I^2^)	Heterogeneity Test (*P* value)
Any Drug Resistance	Overall	36	31 (24, 38)	2045, 7480	97.65	<0.01
	**Stratified by Years**					
	2000–2015	21	36.44 (28.3–44.99)	1605/5024	97.16	<0.01
	2016–2020	15	24.01 (14.12–35.51)	440/2456	97.37	<0.01
	**Stratified by Location**					
	North	2	12.2 (8.19–16.84)	29/230	–	–
	Center	14	42.07 (29.82–54.83)	1124/3554	98.05	<0.01
	East	2	23.79 (17.9–30.21)	45/187	–	–
	West	5	22.86 (4.46–49.48)	123/711	98.10	<0.01
	South	9	33.54 (17.34–51.98)	348/1175	97.62	<0.01
	Different Parts	4	15.6 (6.07–28.42)	376/1623	96.90	<0.01
	**Stratified by DST methods**					
	Proportion Method	24	31.44 (23.6–39.84)	1634/5931	97.69	<0.01
	Double method	12	30.49 (16.17–47)	411/1549	97.73	<0.001
Mono drug Resistance	Overall	26	15 (10, 22)	940/5357	97.33	<0.001
	**Stratified by Years**					
	2000–2015	16	36.44 (28.30–44.99)	820/4003	97.16	<0.001
	2016–2020	5	24.01 (14.12–35.51)	120/1354	97.37	<0.001
	**Stratified by Location**					
	North	2	9.8 (6.19–14.08)	23/230		–
	Center	11	25.39 (13.77–39.08)	684/2527	97.79	<0.001
	East	2	15.44 (10.54–21.04)	32/187		–
	West	4	5.67 (0.53–15.08)	38/661	93.33	<0.001
	South	4	12.45 (0.60–33.95)	40/446	94.90	<0.001
	Different Parts	4	8.66 (6.75–10.78)	123/1370		<0.001
	**Stratified by DST methods**					
	Proportion Method	18	17.86 (10.52–26.60)	854/4459	97.76	<0.001
	Double method	8	10.48 (4.05–19.26)	86/962	92.84	<0.001
Multi-drug Resistance	Overall	40	6 (4, 8)	529/7903	91.96	<0.001
	**Stratified by Years**					
	2000–2015	21	6.92 (4.02–10.47)	192/3429	93.44	<0.001
	2016–2020	19	5.53 (3.38–8.12)	177/3602	88.17	<0.001
	**Stratified by Location**					
	North	2	0.25 (0.00–1.69)	1/230	–	–
	Center	16	7.71 (4.12–12.22)	310/4239	98.08	<0.001
	East	2	2.34 (0.49–5.20)	5/187	–	–
	West	5	6.00 (1.67–12.48)	39/793	96.15	<0.001
	South	9	8.11 (4.19–12.48)	103/1253	95.58	<0.001
	Different Parts	7	5.35 (2.11–9.82)	71/1255	96.25	<0.001
	**Stratified by DST methods**					
	Proportion Method	27	6.45 (4.08–9.28)	398/5775	92.95	<0.001
	Double method	13	6.03 (3.06–9.83)	131/2182	89.56	<0.001
Isoniazid	Overall	38	12 (8, 16)	1029/7391	95.91	<0.001
	**Stratified by Years**					
	2000–2015	21	14.49 (10.13–19.45)	352/4301	94.15	<0.001
	2016–2020	17	9.42 (4.97–15.02)	229/2946	95.19	<0.001
	**Stratified by Location**					
	North	2	3.23 (1.17–6.08)	8/230	–	–
	Center	14	16.67 (6.59–27.23)	435/2955	95.94	<0.001
	East	2	5.01 (2.20–8.73)	10/187	–	–
	West	5	4.52 (1.07–9.92)	41/891	89.44	<0.001
	South	8	15.55 (6.59–27.23)	123/917	94.0.13	<0.001
	Different Parts	9	12.03 (4.27–22.88)	412/2211	97.48	<0.001
	**Stratified by DST methods**					
	Proportion Method	27	12.16 (7.95–17.08)	881/6060	98.63	<0.001
	Double method	7	11.94 (5.37–20.53)	148/1331	97.71	<0.001
Rifampin	Overall	34	11 (8, 16)	589/4896	95.15	<0.001
	**Stratified by Years**					
	2000–2015	19	14.14 (8.53–20.82)	352/4301	94.80	<0.001
	2016–2020	15	8.10 (4.22–13.01)	173/2513	93.55	<0.001
	**Stratified by Location**					
	North	2	1.13 (0.04–3.18)	4/230	–	–
	Center	11	14.75 (7.69–23.52)	229/1421	93.75	<0.001
	East	1	8.54 (3.5–16.8)	7/82	–	–
	West	5	2.71 (0.28–7.05)	30/891	88.57	<0.001
	South	8	21.15 (10.2–34.68)	225/1165	96.38	<0.001
	Different Parts	7	8.66 (3.73–15.27)	94/1255	91.89	<0.001
	**Stratified by DST methods**					
	Proportion Method	22	13.27 (7.89–19.73)	419/3427	96.08	<0.001
	Double method	12	8.27 (3.87–14.03)	170/1617	92.35	<0.001
Ethambutol	Overall	26	8 (4, 11)	363/ 34,985	94.21	<0.001
	**Stratified by Years**					
	2000–2015	14	10.38 (5.51–16.48)	215/1563	92.40	<0.001
	2016–2020	12	4.82 (1.91–8.85)	118/2196	93.57	<0.001
	**Stratified by Location**					
	North	1	0.00 (0.00–2.07)	0/176	–	–
	Center	9	15.51 (7.10–26.30)	213/1550	95.89	<0.001
	East	2	3.19 (0.99–6.36)	6/187	–	–
	West	4	4.52 (0.04–13.98)	25/605	93.20	<0.001
	South	4	10.19 (3.26–20.17)	49/590	91.82	<0.001
	Different Parts	6	2.97 (1.15–5.49)	40/1137	73.97	<0.001
	**Stratified by DST methods**					
	Proportion Method	20	6.84 (3.83–10.75)	263/3465	93.13	<0.001
	Double method	6	10.54 (1.3–26.26)	70/780	96.77	<0.001
Streptomycin	Overall	23	18 (12, 24)	552/3141	94.50	<0.001
	**Stratified by Years**					
	2000–2015	14	23.10 (16.64–3025)	390/1717	90.84	<0.001
	2016–2020	9	10.82 (4.98–18.45)	162/1424	94.35	<0.001
	**Stratified by Location**					
	North	2	8.12 (4.82–12.12)	19/230	–	–
	Center	9	24.74 (13.58–37.52)	317/1550	96.22	<0.001
	East	2	20.12 (14.62–26.23)	39/187	–	–
	West	2	16.66 (12.34–21.48)	44/260	–	–
	South	4	17.56 (7.17–31.1)	51/321	89.13	<0.001
	Different Parts	4	10.27 (1.38–25.47)	82/755	96.68	<0.001
	**Stratified by DST methods**					
	Proportion Method	18	18.45 (12.86–24.77)	470/2814	93.80	<0.001
	Double method	5	15.69 (2.12–37.64)	82/489	96.73	<0.001
HRES	overall	8	3 (1, 5)	39/1022	80.18	<0.001
HRE	overall	9	2 (0, 5)	29/1116	87.97	<0.001
HRS	overall	8	3 (0, 8)	33/836	91.73	<0.001
HR	overall	26	6 (4, 9)	164/4141	92.03	<0.001
	**HR Stratified by Years**					
	2000–2015	14	7.16 (3.67–11.61)	164/1932	91.32	<0.001
	2016–2020	12	5.59 (2.37–9.94)	12/2209	92.39	<0.001
	**HR Stratified by DST methods**					
	Proportion Method	18	6.77 (3.54–10.87)	199/2823	92.75	<0.001
	Double method	8	5.66 (2.15–10.55)	71/1514	90.40	<0.001
	**Stratified by Location**					
	North	1	0.57 (0.01–3.12)	1/176	–	–
	Center	11	7.44 (3.41–12.74)	140/2087	93.05	<0.001
	East	1	4.88 (1.34–12.02)	4/82	–	–
	West	4	4.52 (0.92–10.34)	31/743	88.99	<0.001
	South	5	8.49 (0.99–21.38)	54/699	95.08	<0.001
	Different Parts	4	6.31 (0.42–17.29)	40/550	93.18	<0.001
Pyrazinamide	Overall	8	10 (2, 23)	40/496	95.27	<0.001
	**HR Stratified by Years**					
	2000–2015	7	3.39 (1.40–6.07)	37/308	73.15	<0.001
	2016–2020	1	0 (0–2.25)	34/188	95.27	<0.001
	**Stratified by Location**					
	North	0	–	0/0	–	–
	Center	2	7.27 (4.37–10.8)	21/266		
	East	0	–	0/0	–	–
	West	1	24.11 (16.53–33.10)	27/112		
	South	0	–	0/0	–	–
	Different Parts	2	4.3 (2.15–7.08)	23/280	–	–
	**HR Stratified by DST methods**					
	Proportion Method	7	3.39 (1.40–6.07)	10/196	73.15	<0.001
	Double method	1	0 (0–2.25)	61/462	–	–

CI, confidence interval; n, number of events (drug resistance); N, total number of new cases and retreatment cases from the included studies**;** Double method, Proportion methods plus other methods; DST, drug susceptibility testing; HRES, be resistant to isoniazid + rifampicin + streptomycin + ethambutol; HRE, be resistant to isoniazid + rifampicin + ethambutol; HRS, be resistant to isoniazid + rifampicin + streptomycin; HR, be resistant to isoniazid + rifampicin.

For retreatment cases, resistance was most common against isoniazid (19 %), followed by rifampin (12 %), streptomycin (12 %), and ethambutol (0 %) (Table 2). The summarized prevalence of any-drug resistance, mono-drug resistance, and MDR among retreatment cases in the study population were found to be 68 % (95 % CI 58 %–78 %), 19 % (95 % CI 7 %–34 %), and 28 % (95 % CI 15 %–43 %), respectively (Table 2).

**Table 2:** Prevalence of Drug-Resistant TB Among Retreated Cases in Iran.

Variables	Number of studies	n/N	Prevalence of Drug Resistance [95 % CI]	p-value	Heterogeneity Test (I^2^)
Any drug resistance	6	644, 2562	68 [58], [78]	<0.01	83.53
Mono drug resistance	3	180, 1605	19 [7], [34]	<0.01	91.03
Multi drug resistance	7	340, 2624	28 [15], [43]	<0.01	93.22
Isoniazid resistance	4	281, 1643	19 [2], [46]	0.01	88.09
Rifampicin resistance	3	6, 504	12 [3], [25]	<0.01	0.00
Streptomycin resistance	3	6, 504	12 [3], [25]	<0.01	0.00
Ethambutol resistance	3	1, 504	0 [0, 7]	0.67	4.70

CI, confidence interval; n, number of events (drug resistance); N, total number of retreatment cases from the included studies.

### 3.4 Second-line Anti-TB drug resistance

**Fig. 5:**
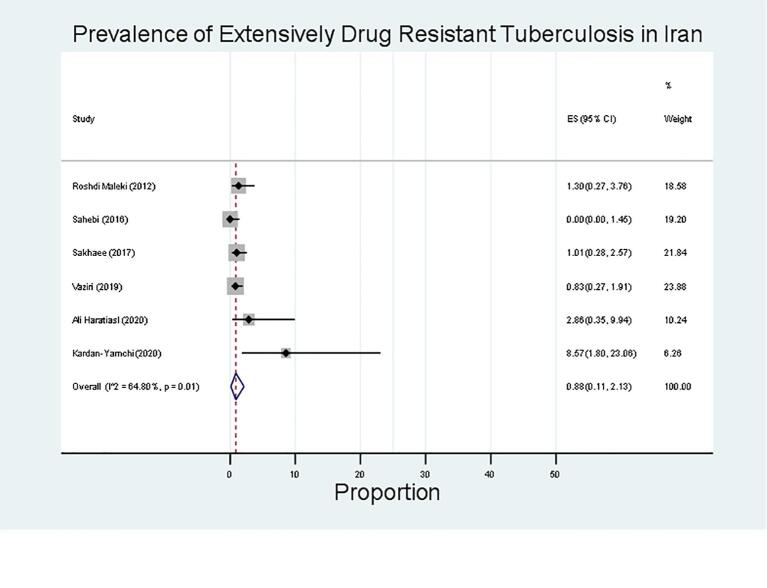
Prevalence of Extensively Drug Resistant TB among new cases in Iran.

Resistance was most common against ethionamide (50 %; 95 % CI, 18–81; 145/313), followed by kanamycin (12 %; 95 % CI, 3–24; 57/861), capreomycin (4 %; 95 % CI, 2–6; 21/430), amikacin (2 %; 95 % CI, 1–5; 11/265), and ofloxacin (4 %; 95 % CI, 0–11; 24/695) (Table 3). The prevalence of any-drug resistance, MDR, pre-extensively drug-resistant (pre-XDR), and extensively drug-resistant (XDR) among new cases was found to be 60 % (95 % CI, 30–86), 10 % (95 % CI, 4–18), 8 % (95 % CI, 0–5), and 1 % (95 % CI, 0–2), respectively. Fig. 5 shows the forest plot of the *meta*-analysis of XDR in TB cases.

**Table 3:** Prevalence of Second line Drug-Resistant TB in Iran.

Variables	N. Studies (n, N)			
		**Proportion (95 % CI)**	**P-value**	**Heterogeneity Test** **(I^2^)**
Any Drug Resistance	5 (335, 965)	60 (30, 86)	<0.01	98.73
Multidrug resistance	9 (110, 1950)	10 (4, 18)	<0.01	95.69
Extensively drug-resistant	5 (17, 1589)	1 (0, 2)	0.01	64.80
Pre-Extensively Drug Resistant	2 (22, 711)	8 (0, 25)	0.04	93.44
Amikacin	1 (11, 265)	2 (1, 5)	<0.01	0.00
Kanamycin	5 (57, 861)	12 (3, 24)	<0.01	94.12
Ethionamide	3 (145, 313)	50 (18, 81)	<0.01	97.10
Ofloxacin	2 (24, 695)	4 (0, 11)	0.01	89.08
Capreomycin	1 (21, 430)	4 (2, 6)	<0.01	0.00

CI, confidence interval; n, number of events (drug resistance); N, total number of retreatment cases from the included studies.

## 4 Discussion

Tuberculosis (TB) remains a significant global health challenge, with millions of people infected and hundreds of thousands of deaths each year. Despite international efforts, the reduction targets for TB incidence and mortality set for 2020 were only partially achieved, emphasizing the persistent threat of TB as a public health problem [59]. This study provides an updated review of first- and second-line drug resistance among new and previously treated TB patients in Iran over the past two decades.

Isoniazid is a crucial first-line anti-TB drug, and resistance to it can compromise treatment success and increase the risk of developing multidrug-resistant TB (MDR-TB) [60], [61]. Rifampin, another essential first-line drug, is a potent sterilizing agent for TB treatment. Rifampin resistance is a key indicator for MDR-TB and poses significant challenges in patient management [62]. Cases resistant to both isoniazid and rifampin can lead to treatment failure and the emergence of MDR-TB [63]. This study found that between 2000 and 2020 in Iran, 12 % of new cases were resistant to isoniazid, while 11 % were resistant to rifampin. Among retreatment cases, 19 % were resistant to isoniazid, and 12 % were resistant to rifampin. Globally, the prevalence of isoniazid resistance among new TB patients is 10.7 %, while it is 27.2 % among previously treated cases [64]. The observed isoniazid resistance rate in Iran (12 %) was lower than that reported in some African countries like Benin (27.9 %) [65] and Ethiopia (15.62 %) [66] but similar to China (12.0 %) [67]. Rifampin resistance rates in this study (11 %) align with international data across different regions [68], [69]. Notably, the rate of any drug resistance among new and previously treated TB cases in Iran was 31 % and 68 %, respectively, suggesting a high rate of acquired resistance to anti-TB medications. This phenomenon may be attributed to factors such as inappropriate prescription practices, drug supply issues, poor drug quality, and high treatment failure rates [61], [70], [71], [72].

MDR-TB is a growing concern that poses a significant challenge to the prevention and control of infectious diseases [73]. The prevalence of MDR-TB among new cases in this study was 6 %, higher than the global (3.4 %) and national average (2.84 %) [74]. This indicates that the burden of MDR-TB among new cases may be underestimated, necessitating increased efforts in detection and treatment. While two previous systematic reviews [6], [7] on TB drug resistance in Iran reported similar findings for new patients, the prevalence of MDR-TB among retreated patients decreased from 33.7 % (Nasiri et al., 2014) [6] to 28 % (present study). However, the rate of MDR-TB among retreated cases in this study was higher than reported in several African countries [66], [75], [76].

Streptomycin resistance was the most prevalent among first-line drugs, affecting 18 % of patients in this study. Conversely, Khademi et al. [7] reported that pyrazinamide was the most resistant anti-TB drug. These variations may stem from differences in drug susceptibility testing methodologies, particularly the drug concentrations used [77].

Among second-line drugs, ethionamide exhibited the highest resistance rate (50 %), followed by kanamycin (12 %), capreomycin (4 %), amikacin (2 %), and ofloxacin (4 %). The global surveillance and treatment of XDR-TB is wholly crucial [78]. The prevalence of any drug resistance, MDR, pre-extensively drug-resistant (pre-XDR), and extensively drug-resistant (XDR) among new cases was 60 %, 10 %, 8 %, and 1 %, respectively. The prevalence of XDR-TB in this study aligns with findings from Khademi et al. [7] (0.9 %), but the pre-XDR prevalence (8 %) is a cause for concern, as it suggests the potential development of XDR cases in Iran.

The study revealed geographic disparities in drug resistance rates, with higher rates observed in central and southern Iran. The central region had the highest resistance rates, possibly due to the concentration of published studies, the referral of drug-resistant MTB isolates to Tehran (the capital), and a higher number of immigrants, particularly Afghans, who have a higher rate of resistance [19].

Despite a decreasing trend in drug resistance from 2000 to 2020, the study highlighted a significant gap between confirmed cases and drug resistance testing in TB laboratories in Iran. This suggests missing drug resistance data and underscores the need for better screening and diagnosis of suspected TB cases [79]. Irregular and incomplete TB treatment and inadequate screening for suspected patients are key factors contributing to drug resistance development and distribution [79].

## 5 Conclusion

In summary, this study reveals that although new and previously treated TB patients in Iran exhibit high levels of resistance to various anti-TB drugs, there has been a promising downward trend in drug resistance rates and TB incidence over the past two decades. However, several critical considerations emerge from these findings.

Firstly, there remains a substantial gap between confirmed TB cases and those who undergo drug resistance testing. This discrepancy highlights the need for improved diagnostic facilities and expanded drug susceptibility testing to cover all confirmed TB cases. Bridging this gap is essential for achieving a sustained decline in TB incidence and drug resistance trends as part of TB control efforts.

Secondly, the study identifies regional variations in drug resistance rates, with some areas exhibiting notably higher rates than others. These disparities emphasize the importance of the lab capacity and staff experience, tailored interventions and resource allocation to address the specific challenges posed by drug-resistant TB in different regions.

Lastly, Iran's proximity to countries with higher TB incidence rates underscores the need for heightened vigilance and cooperation in TB control efforts. Collaborative strategies and information sharing with neighboring nations can contribute to a more comprehensive approach to TB management.

In conclusion, while challenges persist, the overall trajectory of declining TB incidence and drug resistance trends in Iran is encouraging. Continued investment in diagnostic infrastructure, regional-specific interventions, and international collaboration are crucial for sustaining and accelerating progress in TB control and reducing drug resistance rates.

## 6 Limitations

Several limitations should be considered in this analysis, including the uneven representation of Iranian provinces in the included studies, potential heterogeneity between studies, and the inability to analyze the impact of factors such as age, sex, ethnicity, nationality (Iranian vs. Afghan), socioeconomic status, and lifestyle on drug resistance prevalence.

Ethical statement

The study protocol was approved by the Health Research Ethics Committee of Ilam University of Medical Sciences (reference no. IR.MEDILAM.REC.1400.006).

## Funding

Not applicable.

## CRediT authorship contribution statement

**Sara Abbasian:** Writing – original draft, Conceptualization. **Hamid Heidari:** Writing – review & editing, Writing – original draft, Conceptualization. **Danyal Abbasi Tadi:** Software, Methodology, Formal analysis. **Jalil Kardan-Yamchi:** Writing – review & editing, Data curation. **Asieh Taji:** Investigation, Validation. **Atieh Darbandi:** Methodology, Data curation. **Parisa Asadollahi:** Writing – review & editing. **Abbas Maleki:** Data curation, Writing – review & editing. **Hossein Kazemian:** Writing – review & editing, Supervision, Conceptualization.

## Declaration of competing interest

The authors declare that they have no known competing financial interests or personal relationships that could have appeared to influence the work reported in this paper.
